# Extracorporeal life support following out-of-hospital refractory cardiac arrest

**DOI:** 10.1186/cc9976

**Published:** 2011-01-18

**Authors:** Morgan Le Guen, Armelle Nicolas-Robin, Serge Carreira, Mathieu Raux, Pascal Leprince, Bruno Riou, Olivier Langeron

**Affiliations:** 1 Department of Anesthesiology and Critical Care, Centre hospitalo-universitaire (CHU) Pitié-Salpêtrière, Assistance Publique-Hôpitaux de Paris (APHP), Université Pierre et Marie Curie-Paris 6, 47-83 Boulevard de l'Hôpital, F-76651 Paris Cedex 13, France; 2 Department of Cardio-thoracic Surgery, CHU Pitié-Salpêtrière, APHP, Université Pierre et Marie Curie-Paris 6, 47-83 Boulevard de l'Hôpital, F-76651 Paris Cedex 13, France; 3 Department of Emergency Medicine and Surgery, CHU Pitié-Salpêtrière, APHP, Université Pierre et Marie Curie-Paris 6, 47-83 Boulevard de l'Hôpital, F-76651 Paris Cedex 13, France

## Abstract

**Introduction:**

Extracorporeal life support (ECLS) has recently shown encouraging results in the resuscitation of in-hospital (IH) refractory cardiac arrest. We assessed the use of ECLS following out-of-hospital (OH) refractory cardiac arrest.

**Methods:**

We evaluated 51 consecutive patients who experienced witnessed OH refractory cardiac arrest and received automated chest compression and ECLS upon arrival in the hospital. Patients with preexisting severe hypothermia who experienced IH cardiac arrest were excluded. A femorofemoral ECLS was set up on admission to the hospital by a mobile cardiothoracic surgical team.

**Results:**

Fifty-one patients were included (mean age, 42 ± 15 years). The median delays from cardiac arrest to cardiopulmonary resuscitation and ECLS were, respectively, 3 minutes (25th to 75th interquartile range, 1 to 7) and 120 minutes (25th to 75th interquartile range, 102-149). Initial rhythm was ventricular fibrillation in 32 patients (63%), asystole in 15 patients (29%) patients and pulseless rhythm in 4 patients (8%). ECLS failed in 9 patients (18%). Only two patients (4%) (95% confidence interval, 1% to 13%) were alive at day 28 with a favourable neurological outcome. There was a significant correlation (*r *= 0.36, *P *= 0.01) between blood lactate and delay between cardiac arrest and onset of ECLS, but not with arterial pH or blood potassium level. Deaths were the consequence of multiorgan failure (*n *= 43; 47%), brain death (*n *= 10; 20%) and refractory hemorrhagic shock (*n *= 7; 14%), and most patients (*n *= 46; 90%) died within 48 hours.

**Conclusions:**

This poor outcome suggests that the use of ECLS should be more restricted following OH refractory cardiac arrest.

## Introduction

Out-of-hospital (OH) cardiac arrest remains an important cause of unexpected death in developed countries. It still has a low survival rate, despite access to improved emergency medical care, the spread of automatic defibrillation [1] and regularly updated international guidelines [2]. Recent studies have indicated unchanged or slightly better survival rates after OH cardiac arrest over the past decades [3,4]. Initial rhythm and cardiac origin are independent predictors of successful cardiopulmonary resuscitation (CPR), with better outcomes related to a shockable rhythm, such as ventricular fibrillation, than asystole [5,6]. Survival rate rapidly decreases with time and refractory cardiac arrest, defined as persistence of circulatory arrest despite more than 30 minutes of appropriate CPR, is usually considered a condition associated with no survival [7], except in some particular conditions such as hypothermia [8].

Extracorporeal life support (ECLS) has been suggested as a therapeutic option in refractory cardiac arrest since 1976 [9]. However, the use of this technique has remained limited to hypothermic cardiac arrest and those cases occurring during the perioperative period of cardiothoracic surgery, mainly because the results of the initial trials were disappointing [10,11]. The ease of use of more recent miniaturized ECLS devices has permitted a wider use of the technique. Encouraging results have been published recently by several teams in France, Taiwan, Japan and the United States [12-16]. In these studies, most cardiac arrests occurred in the hospital, and survival with good neurological outcome has been observed in up to 20% to 30% of cases [12-16]. Therefore, ECLS has been assigned a low-grade recommendation in recent guidelines for in-hospital (IH) cardiac arrest [17].

However, the good results obtained in IH cardiac arrest should not be extrapolated to OH cardiac arrest, mainly because there may be a longer delay in ECLS initiation [18]. Our primary aim was to review the use of ECLS for OH refractory cardiac arrest.

## Materials and methods

This prospective observational study received approval from our institutional review board (CPP Pitié-Salpêtrière 2008/0701, Paris, France). Informed consent was waived because of life-threatening emergencies and the absence of any therapeutic alternative. Information was delivered to the relatives of the patient (or to the patient in cases of survival) after inclusion as appropriate in a life-threatening context.

### Patients

Over a 32-month period (from January 2008 to August 2010), all patients who were referred to our intensive care unit (ICU) for OH refractory cardiac arrest were eligible for enrollment into this study. They were included prospectively and consecutively if the following criteria were met: (1) witnessed OH cardiac arrest; (2) refractory cardiac arrest, defined as the absence of a return of spontaneous circulation (ROSC) after 30 minutes of CPR; (3) CPR was pursued until the patient's arrival at our ICU; (4) a mobile cardiothoracic surgery team was available; and (5) a lack of known, severe comorbidities that should have precluded admission into an ICU. Patients who experienced IH cardiac arrest were excluded, as well as patients who were severely hypothermic (body temperature <32°C) before CPR. Young children (<30 kg) were not included because our institution exclusively takes care of adults and because specific sizes of paediatric cannulae were not available. Conversely, patients older than 70 years of age were considered ineligible because of the poor expected neurological recovery.

### Protocol

The prehospital emergency medical service (EMS) team performed CPR according to the American Heart Association guidelines [2]. In cases of refractory cardiac arrest, CPR was pursued in the prehospital phase using an automated device (AutoPulse; Zoll Inc., Chelmsford, MA, USA) [19]. In the Paris area, all prehospital physician-staffed emergency units are equipped with an automated chest compression device because France has developed a nationwide program of organ harvesting in non-heart-beating donors. As soon as the EMS team determined that initial CPR had failed, they immediately alerted our ICU through the emergency unit regulating centre to organize the patient admission and to ensure the availability of the mobile cardiothoracic surgery team. This unit works in our hospital and includes a surgeon, a resident in surgery and a technician, together with full equipment required to set up emergency ECLS anywhere. This mobile cardiothoracic surgery team has had extensive experience in our hospital and in our city [12,20,21]. During transfer to the hospital, resuscitation was continued without stopping at any moment.

At the admission to the ICU, the absence of ROSC and the absence of a heartbeat were checked before engaging in the procedure. Then mechanical ventilation and an automated chest compression device were used until the start of ECLS. ECLS was established surgically with peripheral femorofemoral cannulation. The equipment used included heparinized polyvinyl chloride tubing, a membrane oxygenator (Quadrox Bioline; Jostra-Maquet, Orleans, France) and venous and arterial femoral cannulae (Biomedicus Carmed; Medtronic, Boulogne-Billancourt, France) inserted surgically. An oxygen-air blender (Sechrist; Sechrist Industries, Anaheim, CA, USA) was used to supply the membrane oxygenator. Pump flow was initially set at 3 to 4 L min^-1^, and then arterial and central venous catheters were inserted to continuously measure arterial blood pressure and allow frequent blood sampling. To avoid limb ischemia, an anterograde reperfusion catheter for distal limb perfusion was inserted. Objectives to optimize organ perfusion were partial pressure of oxygen (PaO_2_) >100 mmHg, normocapnia and arterial blood pressure >60 mmHg with administration of fluids, blood transfusion to achieve a hematocrit level >35% or vasopressive drugs (norepinephrine or epinephrine). Mild hypothermia (target body temperature 33°C to 35°C) was maintained during the first 24 hours using external cooling (pulsed-air blanket), and neuromuscular blocking agents with sedatives were administered [22]. Minimum lung ventilation was maintained to avoid pulmonary collapse during ECLS with a tidal volume of 4 to 5 mL kg^-1^, a respiratory rate of 6 breaths minute^-1 ^and positive end-expiratory pressure of 5 cmH_2_O. To avoid coagulation in the membrane oxygenator, unfractionated heparin was intravenously administered during ECLS with repeated control to maintain the activated clotting time ratio >2.0, but this administration was postponed in most patients because of major coagulation abnormalities. An inhibitor of the proton pump was systematically administered to prevent upper gastrointestinal bleeding. The possible cause of cardiac arrest was immediately investigated with regular and repeated cardiac troponin I (troponin Ic) assays, transthoracic or transoesophageal echocardiography and an electrocardiogram as soon as electrical activity was present. In this context, patients with strong indications of acute myocardial infarction were transported to the catheter laboratory with ECLS so that percutaneous coronary angiography and any appropriate invasive treatments could be performed [23]. Sedation was stopped after a 24-hour period of mild hypothermia, and then the patient's neurological (clinical examination and eventually electroencephalogram) and infectious status were checked. Withdrawal from ECLS required an echocardiographic assessment of myocardial function (left ventricular ejection fraction >50%) and an arterial blood PaO_2_-to-FiO_2 _(fraction of inspired oxygen) ratio >150 mmHg. The pump flow was progressively reduced to check for the absence of any deterioration in hemodynamic status. The possibility of the use of a ventricular assistance device or heart transplantation was examined if irreversible damage in myocardium function was diagnosed with unsuccessful weaning from ECLS despite a favourable neurological outcome. Discontinuation of ECLS was based upon evidence of multiple organ failure (MOF), massive bleeding or brain death. Any ECLS-associated complications were carefully monitored.

### Measurements

The following variables were recorded according to the Utstein style [24]: age, sex, cardiovascular risk factors, delays from collapse to basic CPR, advanced CPR, installation of automated chest compression device, arrival at the ICU and installation of ECLS, initial cardiac rhythm, use of vasopressor and defibrillation during initial CPR and supposed cause of cardiac arrest. The patient's end tidal CO_2 _(E_T_CO_2_) level during CPR and before ECLS was also recorded [25]. During CPR, signs of life (that is, respiratory gasps, movements) were noted.

The following biological measurements were performed before ECLS: arterial blood gas analysis, blood lactate (normal range, <1.8 mM L^-1^), serum creatinine, blood potassium, fibrinogen and prothrombin activity. Troponin Ic level (normal range, <0.15 μg L^-1^) (Stratus Autoanalyser; Dade-Behring, Paris La Défense, France) and protein S100β level (normal range, <0.10 μg L^-1^; LIA-mat 300 analyzer, Byk-Sangtec France Laboratories, Le Mée sur Seine, France) were also measured [26]. The evolution of arterial pH and blood lactate levels was recorded after 1 to 2 hours of ECLS, and the following variables were calculated: change in arterial pH, blood lactate clearance expressed as a percentage of initial values and the number of patients with blood lactate clearance less than or equal to -10% as previously described [27].

The final outcome was determined at day 28, and the Glasgow Outcome Scale score was determined at 6 months. The Glasgow Outcome Scale comprises the following scores: 1, death; 2, persistent vegetative state; 3, severe disability (minimally conscious state, severe motor deficit, aphasia and need for continuous help); 4, moderate disability; and 5, good recovery.

Since the start of our study (January 2009), French guidelines for the indications for the use of ECLS in refractory cardiac arrest have been published [28]. These guidelines consider the following variables to determine whether ECLS is indicated following OH cardiac arrest: duration of no flow (≤5 min), duration of low flow (≤100 min) and E_T_CO_2 _level (≥10 mmHg), at least in nonhypothermic patients and in patients without life signs during ongoing CPR (Additional file 1) [28]. Thus we assessed whether the main criteria (no flow, low flow and E_T_CO_2 _level) recommended in these guidelines were followed during our study and if the whole algorithm was respected.

### Statistical analysis

Data are expressed as means ± SD or medians (25th to 75th interquartile range (IQR)) for non-Gaussian variables (Kolmogorov test). Categorical variables are given as percentages with their 95% confidence intervals. Comparison between two groups was performed using Student's *t*-test, the Mann-Whitney *U *test or Fisher's exact test as appropriate. Correlation between two variables was performed using least squares linear regression analysis. All *P *values were two-tailed, and a value less than 0.05 was considered significant. Statistical analysis was performed using NCSS 6.0 software (Statistical Solutions Ltd, Cork, Ireland).

## Results

During the study period, we performed ECLS in 59 patients who had experienced refractory cardiac arrest. Three patients who had experienced IH cardiac arrest, as well as five patients with severe hypothermia (24.5°C ± 1.8°C) before cardiac arrest, were excluded. Thus, 51 patients were included in the study. The main characteristics of our population are shown in Table 1. The supposed causes of cardiac arrest were cardiac (*n *= 44, 86%), trauma (*n *= 3, 6%), drug overdose (*n *= 2, 4%), respiratory (*n *= 1, 2%) and electrocution (*n *= 1, 2%). Only one patient had signs of life during CPR before ECLS.

**Table 1:** Main characteristics of the patients (*n *= 51)

Variable	Values	Range
Mean age ± SD, yr	42 ± 15	13-70
Men, *n *(%)	46 (90%)	
Women, *n *(%)	5 (10%)	
Comorbidity, *n *(%)		
Hypertension	6 (12%)	
Diabetes mellitus	3 (6%)	
Ischemic heart disease	11 (20%)	
Other cardiac disease	10 (20)	
Site of cardiac arrest, *n *(%)		
Home	19 (37%)	
Work	6 (12%)	
Public	20 (39%)	
Sport	6 (12)	
Initial rhythm, *n *(%)		
Ventricular fibrillation	32 (63%)	
Asystole	15 (29%)	
Pulseless rhythm	4 (8%)	
Defibrillation		
Patients receiving shock, *n *(%)	37 (72%)	
Number of shocks, *n *(25th to 75th IQR)	4 (2 to 6)	1-20
Epinephrine		
Patients receiving epinephrine, *n *(%)	51 (100%)	
Dose (mg)	13 (10 to 20)	2-100
Mean end tidal CO_2 _± SD, mmHg	22 ± 12	0-50
Delay, median (25th to 75th IQR)		
Fall to basic CPR, min	3 (1 to 6)	0-22
Fall to advanced CPR, min	12 (5 to 23)	0-40
Fall to automated CPR, min	41 (30 to 55)	15-110
Fall to ICU admission, min	90 (65 to 115)	48-175
Fall to ECLS onset, min	120 (102 to 149)	75-195
Biological measurement		
Arterial pH	6.93 ± 0.17	6.56-7.25
Mean blood lactate ± SD, mM L^-1^	19.9 ± 6.7	7.7-40.8
Mean arterial bicarbonate ± SD, mM L^-1^	16.5 ± 12.1	1.9-58.7
Mean PaO_2 _± SD, mmHg	135 ± 129	6-489
Mean PaCO_2 _± SD, mmHg	69 ± 25	19-128
Mean blood potassium ± SD^a^, mM L^-1^	5.1 ± 1.7	2.7-10.5
Mean serum creatinine ± SD, μM L^-1^	129 ± 30	51-275
Mean prothrombin time, %	39 ± 16	11-66
Median fibrinogene, g L^-1 ^(25th to 75th IQR)	1.3 (<0.6 to 1.6)	<0.6-3.6
Mean hemoglobin ± SD, g L^-1^	109 ± 25	59-169
Median troponin Ic, μg L^-1 ^(25th to 75th IQR)	3.98 (0.93 to 85.5)	0-669.0
Median protein S100^b^, μg L^-1 ^(25th to 75th IQR)	4.2 (2.4 to 10.4)	0-36.0

CPR, cardiopulmonary resuscitation; ECLS, extracorporeal life support; ICU, intensive care unit; IQR, interquartile range; PaCO_2_, partial pressure of carbon dioxide; PaO_2_, partial pressure of oxygen; troponin Ic, cardiac troponin I.

^a^Blood potassium could not be measured in four patients because of hemolysis; ^b^Protein S100 was measured in only 27 patients.

ECLS flow could not be established in nine (18%) patients. These patients had a more prolonged no-flow duration (medians, 3 minutes (IQR, 0.5 to 6.5) vs. 2.5 minutes (IQR, 1 to 6); *P *= 0.04) and lower mean E_T_CO_2 _levels (9 ± 3 minutes vs. 12 ± 2 minutes; *P *= 0.046) than the remaining patients. In one case, the failure was the consequence of an impossible cannulation of the femoral artery, probably related to an aortic dissection. The remaining failures of ECLS were related to insufficient pump flow despite massive fluid challenge and transfusion. In the remaining 42 patients, the initial mean ECLS output was 3.6 ± 1.8 L min^-1^, providing a mean arterial blood pressure of 67 ± 39 mmHg. During the ECLS procedure, 30 patients (59%) required blood transfusions (median, 4 packed red blood cell units (range, 2 to 6)). Mean body temperature was 34.1 ± 0.9°C.

Twenty (48%) of the 42 patients in whom ECLS was initiated underwent coronary angiography because of clinical or electrical signs suggesting myocardial infarction, but significant coronary abnormalities were noted in only 10 (50%) of these patients. Angioplasty without stenting was performed in one patient, and coronary stents following percutaneous transluminal angioplasty were inserted in seven patients. Coronary spasm was diagnosed in the two remaining patients.

**Figure 1:**
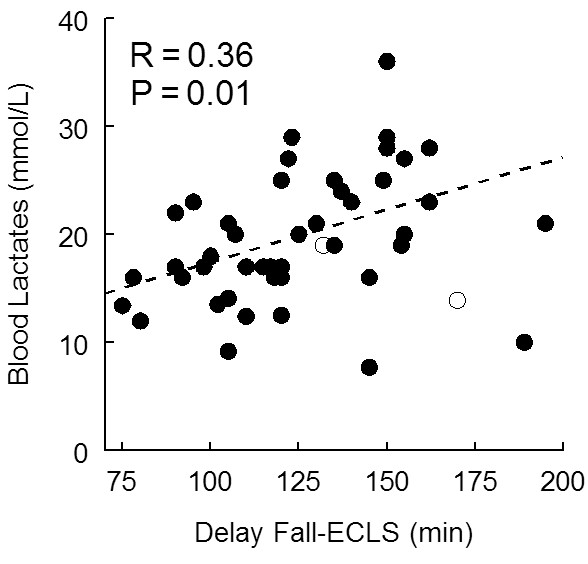
**Relationship between initial blood lactate level and delay between fall and onset of extracorporeal life support (ECLS) (*n *= 48)**.

**Figure 2:**
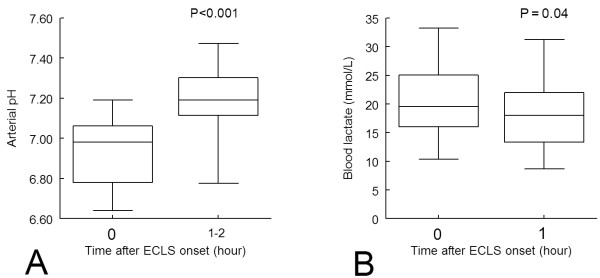
**Kinetic graph of (A) arterial pH and (B) arterial blood lactate during the first hour following extracorporeal life support (ECLS) (*n *= 38)**. Boxplot represents the median, 25th to 75th interquartile range and extreme values.

Arterial blood gas samples at admission showed severe lactic acidosis (Table 1). Initial blood lactate levels were significantly correlated with the OH duration of cardiac arrest until ECLS (*P *= 0.02) (Figure 1). In contrast, the correlations between arterial pH (*r *= 0.05) or blood potassium levels (*r *= 0.23) were not significant. After 1 hour of ECLS, blood lactate levels slightly but significantly decreased, whereas arterial pH markedly increased (Figure 2).

Seventeen (40%) of the 42 patients in whom ECLS was initiated survived after 24 hours of ECLS, but only 5 (12%) survived after 48 hours. At day 28, only two patients were alive, providing a global survival rate of 4% (95% confidence interval, 1% to 13%). The causes of death were refractory MOF (*n *= 23; 45%), brain death (*n *= 10; 20%) and severe haemorrhage (*n *= 7; 14%), with the cause of death in the remaining patients being failure of ECLS (*n *= 9; 18%).

In the first survivor (cardiac cause; ventricular fibrillation; no flow, 1 minute; low flow, 132 minutes; protein S100 level, 1.5 μg L^-1^), withdrawal from ECLS was possible only at day 36 because of severe, prolonged heart failure (left ventricular ejection volume estimated to be 30%), and an implantable automatic defibrillator was inserted. This patient's length of stay in the ICU was 58 days and IH was 187 days. Follow-up at 6 months showed only minor cognitive dysfunction (Glasgow Outcome Scale score 5) but a persistent altered left ventricular ejection fraction (35%). In the second survivor (cardiac cause; ventricular fibrillation; no flow, 0 minutes; low flow, 170 minutes; protein S100 level, 4.5 μg L^-1^), withdrawal from ECLS was possible at day 5. This patient's length of stay in the ICU was 25 days and IH 77 days, with a Glasgow Outcome Scale score 4 at 6 months.

**Figure 3:**
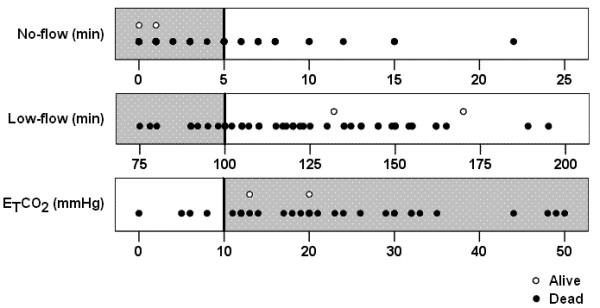
**Distribution of the values of no flow (top), low flow (middle) and end tidal CO**_**2 **_**(E**_**T**_**CO**_**2**_**) (bottom) initial arterial pH, blood lactate and kalemia in the studied population (*n *= 51)**. The gray zones and vertical bars indicate the threshold considered in the French guidelines for no flow (≤5 min), low flow (≤100 min) and E_T_CO_2 _(≥10 mmHg).

Conformity of the cases considering a no-flow period ≤5 minutes was noted in 36 patients (71%), a low-flow period ≤100 min in 14 patients (27%) and E_T_CO_2 _≥10 mmHg in 32 patients (63%). Conformity to the whole algorithm of the French guidelines was seen in eight patients (16%). Figure 3 shows the distribution of values of no flow, low flow, arterial pH, blood lactate and potassium in patients who died and in survivors. Although the two survivors fulfilled the criterion of no flow less than 5 minutes, they did not fulfill the criterion of low flow less than 100 minutes (Figure 3). In an attempt to identify futile ECLS, we compared patients who survived less than 24 hours with those who survived more than 24 hours (*post hoc *comparison). Only E_T_CO_2 _level (means, 18 ± 10 mmHg vs. 29 ± 12 mmHg; *P *= 0.006) and blood lactate clearance during ECLS (medians, 11% (IQR, -24 to 26) vs. -22% (IQR, -26 to -1); *P *= 0.045) were significantly different between patients who survived less than or more than 24 hours.

## Discussion

Our primary objective in this study was to assess the use of ECLS following OH refractory cardiac arrest. In a selected population, we observed 4% survival with good neurological outcomes. Although this survival rate is close to that observed in the general population in France who undergo nonrefractory OH cardiac arrest [29], it represents a low survival rate compared with rates in previous studies of ECLS in IH cardiac arrest.

Refractory cardiac arrest is defined by the lack of ROSC within a period of at least 30 minutes of CPR in the absence of preexisting hypothermia [1,28]. Because this condition is associated with no survival, it is an indication for stopping CPR and declaring the patient dead. It indicates both the absence of the likelihood of restoring cardiac activity and a poor chance of obtaining a good neurological outcome. The introduction of ECLS has created a new paradigm, since refractory cardiac arrest might now be defined only as a function of the possibility of obtaining a good neurological recovery because ECLS supports cardiac function [27]. Recent publications have shown very encouraging results, with 17% to 30% of survivors experiencing a good neurological outcome [12-14,16,30]. Several studies and a meta-analysis have also reported favourable outcome in children [29,31,32]. However, most of these patients experienced IH cardiac arrest. The marked difference in prognosis between IH and OH cardiac arrest has been well recognized but is only partly explained by a shorter treatment delay [18]. Kagawa *et al*. [15] compared IH and OH refractory cardiac arrest treated with ECLS and reported a lower survival rate in OH cardiac arrest (10% vs. 26%). Our results cannot be extrapolated to patients with recurrent cardiac arrest [33] and to those with circulatory failure after ROSC, in whom ECLS might be a therapeutic option. ECLS has also been used successfully in patients with cardiogenic shock before cardiac arrest, particularly in cases of severe drug intoxication [34].

Several factors may explain the low survival rate in OH refractory arrest treated with ECLS. The most important is probably the delay required to start ECLS (that is, low flow), with the minimum being 75 minutes in our study, whereas ECLS was started within 50 minutes in 50% of patients in a previous study [14]. Some studies have reported a relationship between the probability of survival and low-flow duration [14], but some others did not [32]. Part of this delay is unavoidable, but some time could probably be saved by earlier alerting of the system before reaching the 30-minute delay point until diagnosing a refractory cardiac arrest [28]. The no-flow duration may also be crucial, and the best candidates remain those patients who benefit from immediate CPR (that is, 0 no flow). The role of the delay until initiation of advanced CPR may be far less important and was not considered in the French guidelines [28], and the essential role of immediate CPR even without ventilation has been largely confirmed [35]. However, other important factors should be considered, particularly the quality of CPR during ground transportation. The limited number of people available to perform CPR during the prehospital phase and the difficulties associated with transportation are strong arguments for using an automated chest compression device. However, the quality of CPR provided may not be optimal, since this device was demonstrated to have failed to improve survival in a randomized study [19] and may be more heterogeneous between patients than standard CPR.

Victims of refractory cardiac arrest are widely considered potential non-heart-beating organ donors [36]. From an ethical point of view, and beyond the dead organ donor rule [37], it is essential that a clear separation exists between those patients who should be considered for organ donation and whose death is declared and those patients who might benefit from a therapeutic option such as ECLS. The French guidelines tried to help the physician by explaining the contraindications for ECLS in refractory cardiac arrest [28]. Our study suggests that the criteria of no flow ≤5 minutes and E_T_CO_2 _level ≥10 mmHg remain appropriate, although the latter criterion could be considered too liberal when taking into account the lower E_T_CO_2 _level in patients who survive less than 24 hours. In contrast, low flow ≤100 minutes might be too restrictive, since a survivor was observed after no flow 132 minutes (Figure 3) as previously reported [12]. However, because of the low global survival rate, extension of the criteria may not be suitable, and most studies performed in IH cardiac arrest have considered only patients with low flow ≤100 minutes [13,14]. Thus, although the low-flow criteria may remain a matter of debate, more criteria are warranted.

Prolonged CPR leads to severe lactic acidosis and hyperkalemia, and we observed a more severe decrease in pH than that observed in IH cardiac arrest [30]. Müllner *et al*. [38] demonstrated a significant correlation between total duration of cardiac arrest and admission levels of arterial lactate concentration and observed that a lactate level >16.3 mmol L^-1 ^was systematically associated with impaired neurological recovery. However, this result may not apply to patients undergoing ECLS, and although we observed a significant correlation between the duration of low flow and blood lactates (Figure 1), no precise threshold could be used to decide whether to initiate ECLS (Figure 3) as previously noted [12]. Arterial pH and blood potassium level are also potential biological candidates. Nevertheless, the lack of a significant relationship between low-flow duration and these biological variables is not encouraging. The troponin Ic values are probably not useful, since they reflect cardiac injury related to both CPR and the cause of cardiac arrest. The value of protein S100 might be helpful, although our survivors had elevated values. Although most of the early deaths in our study were related to MOF and massive haemorrhage, biological variables exploring hemostasis abnormalities also do not seem very interesting for that purpose. We consider that a large multicentre study with an increased number of survivors using multivariate analysis is mandatory to improve the decision whether to perform ECLS in these patients.

There is growing interest in measuring lactate clearance [26,39]. We observed that ECLS induced a rapid and marked increase in pH but a slight decrease in blood lactate level during the first hours after ECLS (Figure 2). Although there was no significant difference in arterial pH change during ECLS between patients who survived more than 24 hours and those who did not, blood lactate clearance was significantly greater, suggesting that blood lactate clearance may help to decide whether to initiate earlier interruption of futile ECLS.

A potential limitation of a wider use of ECLS in refractory cardiac arrest was the fear that it might lead to the survival of patients with poor neurological recovery and the associated use of costly resources and considerable suffering for the patients and their relatives [28]. Our study confirms that in nonsurvivors, death occurs rapidly because of irreversible MOF or massive haemorrhage. Moreover, most patients with isolated brain injury evolved to brain death and not to a vegetative state. Compared to previous studies [12-15,30], we observed a higher incidence of MOF and massive haemorrhage, probably because of the longer CPR duration, and this finding is reflected by the major haemostasis abnormalities observed before ECLS.

Some limitations in our study deserve consideration. First, the sample size was relatively low. Nevertheless, our study enables us to alert the medical community about the risk of futile resuscitation in most cases of OH refractory cardiac arrest. Second, the absence of a control population of victims of cardiac arrest was ethically justified because the natural evolution of refractory cardiac arrest remains death. Our results may not apply to a paediatric population, since the cause of OH cardiac arrest in children differs markedly from that in adults. Because the respiratory causes are predominant in children, refractory cardiac arrest may indicate that the heart suffered from prolonged anoxia and thus that severe brain damage has occurred.

## Conclusions

ECLS may be an appropriate therapeutic option in patients following OH refractory cardiac arrest, since survival with good neurological outcomes can be observed. However, because the survival rate (4%) remains markedly lower than that in patients with IH refractory cardiac arrest, the indications for ECLS should be restricted to a highly selected population. Further prospective, multicentre studies are needed to define the population with OH refractory cardiac arrest who would benefit from ECLS.

## Key messages

• Extracorporeal life support (ECLS) has shown encouraging results in the resuscitation of in-hospital patients with refractory cardiac arrest.

• We assessed the use of ECLS following out-of-hospital refractory cardiac arrest in 51 patients and observed a low survival rate (4%).

• Further prospective multicentre studies are needed to define the patient population with out-of-hospital refractory cardiac arrest who would benefit from ECLS.

## Abbreviations

CPR: cardiopulmonary resuscitation; ECLS: extracorporeal life support; EMS: emergency medical service; E_T_CO_2_: end tidal carbon dioxide; IH: in-hospital; MOF: multiple organ failure; OH: out-of-hospital; ROSC: return of spontaneous circulation.

## Competing interests

The authors declare that they have no competing interests.

## Authors' contributions

MLG and ANR conceived the study and performed data acquisition, data analysis and interpretation of the data. SC and MR made substantial contributions to the acquisition and interpretation of the data and helped to draft the manuscript. PL conceived the study and was responsible for the ECLS mobile team. BR conceived the study, performed the statistical analysis and wrote the manuscript. OL conceived the study and participated in its design and coordination. All authors read and approved the final manuscript.

## Supplementary Material

Additional file 1 **Algorithm used to decide whether extracorporeal life (ECL) support in treating patients in refractory cardiac arrest (CA) is indicated**. From Riou *et al*. [28]. CPR, cardiopulmonary resuscitation; VT, ventricular tachycardia; VF, ventricular fibrillation; TP, torsades de pointes; E_T_CO_2_, end tidal CO_2 _(measured 20 minutes after the onset of medical CPR). *CPR duration >100 minutes could be accepted in cases of poisoning with cardiac drugs. †Indications accepted by ILCOR. Comorbidities are those which should contraindicate invasive care (for example, admission to the intensive care unit, major surgery, coronary angioplasty). The low-flow duration encompasses basic CPR (witnesses and/or paramedics) and medical CPR. Click here for file
