# Supplementary material for: Extracorporeal life support following out-of-hospital refractory cardiac arrest
Source: Crit Care. 2011 Jan 18;15(1):R29. doi: 10.1186/cc9976 (PMC3222065; doi:10.1186/cc9976)
Supplement: Additional file 1 — Algorithm used to decide whether extracorporeal life (ECL) support in treating patients in refractory cardiac arrest (CA) is indicated. From Riou et al. [28]. CPR, cardiopulmonary resuscitation; VT, ventricular tachycardia; VF, ventricular fibrillation; TP, torsades de pointes; ETCO2, end tidal CO2 (measured 20 minutes after the onset of medical CPR). *CPR duration >100 minutes could be accepted in cases of poisoning with cardiac drugs. †Indications accepted by ILCOR. Comorbidities are those which should contraindicate invasive care (for example, admission to the intensive care unit, major surgery, coronary angioplasty). The low-flow duration encompasses basic CPR (witnesses and/or paramedics) and medical CPR. [file cc9976-S1.DOC]

**Possible indication**

**Uncertainty**

**No indication**

Refractory CA

Cardiac drug Intoxication †

Hypothermia †

(≤ 32°C)

Co-rmorbidities

Signs of life

per-RCP

Assessment of no-flow

duration

0-5 min

Assessment of low-flow duration

> 5 min or no witness

Rhythm

assessment

VT, TP, VF

Asystole

Agonal rhythm

ETCO_2_ ≥ 10 mmHg

**AND** Low-flow ≤ 100 min *

ETCO_2_ < 10 mmHg

**OR**

Low-flow > 100 min

**Additional file 1:** A suggested algorithm to decide whether extracorporeal life support in treating refractory cardiac arrest (CA) is indicated or not. CPR: cardiopulmonary resuscitation ; VT : ventricular tachycardia; VF : ventricular fibrillation; TP : torsades de pointes ; ETCO_2_ : end-tidal CO_2_ (measured 20 min after the onset of medical CPR). *: a CPR duration > 100 min could be accepted in case of poisoning with cardiac drugs. †: indications accepted by ILCOR. Co-morbidities are those which should contra-indicated invasive care (admission into ICU, major surgery, coronary angioplasty for example). The low-flow duration encompasses basic CPR (witness and/or paramedics) and medical CPR. From Riou et al. [28].
